# Reply to a Criticism of Z. C. McElroy, M. D.

**Published:** 1870-03

**Authors:** H. Culbertson

**Affiliations:** Assistant Surgeon U. S. Army, retired, Zanesville, Ohio


ARTICLE II. — *Reply to a Criticism of Z. C. McElroy, M.D., President of the "Muskingum County Medical Society," in Relation to an Article, by the Writer, upon the "Physical Basis of Life,"* by H. CULBERTSON, M.D., Assistant Surgeon U. S. Army, retired, Zanesville, Ohio.

EARLY in December last we read an article on the "Physical Basis of Life," before the above-named society. Its president, the gentleman named, has criticised, in the *Chicago Medical Journal*, certain qualities mentioned in the paper, which we believe to be peculiar to *organic life*, and has attempted to show that these all depend on "form" and "motion."

It may be premised that, if it is not the object of this gentleman to establish a system of medicine, based on "form" and "motion," and not a mere classification, his essay is worthless as a medical production, and deserves no comment as an *iconoclastic* effort. *Classifications* aid greatly in the acquirement of knowledge, but are far different from a SYSTEM based upon assumed or proven principles.

As, however, it is manifest, from a reading of this gentleman's paper, that the object is to inaugurate a SYSTEM OF MEDICINE on two of the qualities of matter, viz.: "form" and "motion," all the other properties of matter are at once ostracized by the very nature of this assumed predicate, "form" and "motion."

To proceed: "Form," from *forma*, "abstractly a figure," (in this connection) relates to contour, the external, of bodies. Thus we speak of the "form" of crystals, of hepatic cells, etc., meaning, thereby, simply their figure. With this plain definition of "form" in view, the objections to the attempt to establish a scientific system of medicine, based, in part, on "form," are:

1st. That as "forms" (organic) are composed of particles, there is a point in the origin of organized bodies, when the matter of which they are composed becomes invisible, naturally and artificially. Hence, to support this form-doctrine, as a life-basis, it is necessary to assume that "form" does obtain behind the visible "form," when it cannot be proved; and this, it is claimed, is unscientific. The outlines of organic "forms" might be this or that, when beyond our inspection; and as the microscopic field is enlarged, units are resolved into compounds, and new features are developed in life previously unsuspected.

But the ultimately visible "granule" of the life-cell, does not possess definite "form." Inclining to curved lines, it is not uniform in its figure, and hence we can predicate no *definite* system of medicine on the *indefinite* "figure" of a germ-life-granule. The diverse "forms" of individual classes of life-cells corroborate this view: Tesselated epithelium is circular, polygonal, or irregular;* the "form" of fat vesicles is globular, oval, or polygonal;† cartilage-cells are irregular in size and shape;‡ and air-cells vary in size and shape, but incline to an angular "form."§ Such diversity in the cell-figure of typical life-structures, refutes the value of any system based on "form."

2nd. The fact that this ultimately visible "form," being actuated by a power to appropriate that which is proper for its maintenance, loses and gains particles continually, and hence, therefore, cannot *strictly* be possessed of the *same* "figure" at these formative and destructive periods of the life-"form;" and the fact that this action is accomplished on particles of life-matter of diverse "form," adds weight to this argument.

3rd. The fact and conclusion that "form" being the result of *force* and *figure*, the basis of this system of medicine, we are precluded from considering the *power* which actuates the "form," as contour is the basis; and the instant we begin to contemplate the *other* qualities of matter, as inertia, divisibility, compressi-

* *Hassall's Mic. Anatomy*, Vol. I, p. 265.

† Ditto, Vol. I, p. 254.

‡ Ditto, Vol. I, p. 307.

§ Ditto, Vol. II, Plate xlviii, Fig. 3.

bility, expansibility, porosity, (but not mobility, by this system,) we are without the pale of this figure-basis, and our consideration of these qualities of matter in general leads to the conclusion that the "form"-basis is inadequate as a scientific basis in medicine, or the act would not have been done.

4th. The fact that all the other qualities of a life-"form" do not emanate "from" *figure*, is an objection, as the basis of a system here should be that from which all the other qualities spring. It would seem probable that definite "form" is not necessary that life-qualities may be manifested, and incorrect to maintain that without "form" none of the physical qualities of matter could be developed in a life-body, the truth being that matter, force, and the qualities of matter *in general*, are required in this relation.

5th. The consideration that figure, being only the exterior, and the basis of this system, histology and pathology become of secondary importance, as the investigation of the interior structure is, by the nature of this *figure*-basis, logically debarred.

6th. The fact that, as physicians, our attention being constantly directed to the *exterior*, by this system, will create in us a tendency to lose sight of the organic *interior*, and thus will we neglect the internal operations of an organic body, and be led to ignore our natural desire to look beyond the "figure" into the *structure.*

7th. The fact that, in organic generation, the ultimately visible germ (which has no uniform figure) develops so many structures having *different* "forms;" and that this diversity cannot be accounted for on any law of "form."

8th. The fact that organic generation takes place by cleavage of "form," or the introduction of the material quality, "divisibility," is an argument against this "form"-basis as a sole foundation in relation to this subject.

9th. The fact that this system ignores causes (is based on figure), and investigates alone effects ("form" is the result of *force*), is prejudicial to progress in medicine, and opposed to our imperative duty, in the cure of disease, to look for causes, and is not equal to our present system, which considers the exterior, the interior, and causes.

9th. The fact that the visible features of the infant are so changed as not to be recognized in the adult, though the last proceeds from the first, is a potent argument against any doctrine based on "form."

10th. The fact that in certain varieties of paralysis the muscular fibres retain their "form," but the *structure* presents fatty degeneration,* leads to the conclusion that although the "form" is unaffected, the *structure* may be, and hence this "figure"-basis does not meet all the circumstances attending such a transformation. And, again, in simple hypertrophy, although the "form" is unchanged, being only enlarged, its mere increase of size (or "form") may lead to disease.

11th. The fact that if "form" be understood to mean "idea" (one of its definitions), a system of medicine based on such a "form," would be founded on an *immaterial* characteristic, and hence would clearly not be established on a "physical basis."

For these reasons, and others which might be given, it is claimed that a scientific system of medicine cannot be established on "form."

The objections to "motion," as the basis of a scientific system of medicine, we believe are as follows, it being understood that "motion," *moteo*, means "change of local position:"

1st. That the test "motion" will not enable us to define the *modus operandi* of medicines, and hence, were it the basis, it would still be necessary to depend on the *experimentum crucis* to solve this point.

2nd. The conclusion that the motion, in an organic body, cannot be so rapid in its molecular action, as to destroy it, by tissue-rending, and hence, that simple "motion" cannot account for molecular death. Thus the most rapid growths, as fungi, developing by millions per minute, reach perfection (yet die soon); and in the human body the impetus of the fluids in disease, over that in health, is not sufficiently great to give rise to maladies by mere velocity. Such rapid development as is seen in the growth of fungi, would destroy many objects in the physical world, and yet does not the fungi.

3rd. The conclusion that, in cholera, and several adynamic diseases, there is rapid waste-"motion," *with lowness of temper-*

* *Gross Surgery*, Vol. I, p. 252, first edition.

*ature*, when in physics *increase* of temperature obtains from augmentation of "motion;" and hence the *reduction* of temperature, observed in cholera, does not comport with one of the laws of physics, and should not, therefore, be the basis of a *physical* system of medicine.

4th. The conclusion that secretion cannot be induced in a glandular organ, while inflamed, (under excessive motion) and until its excitement has been reduced, or from *diminished motion*, results *increased action* (the secretion), is contrary to a law of physics, for *diminished heat* should induce *diminished* motion.

5th. The conclusion that, in certain stages of many diseases, the rapid circulatory "motion" denotes debility, not strength; and yet, by this "motor" theory, the more rapid the motion the greater should be the strength, when the truth is, in the animal body, the slower (within a certain range) the motion, the greater is the strength; and hence the rejection of this "motor"-basis, as not in consonance with another of the laws of physics.

6th. The fact that certain medicines *diminish* motion (*digitalis* on the heart), but *increase* strength, when, on this theory, such agents, in *diminishing* action, should *decrease* strength, is opposed to this "motion"-basis, and another of the laws of physics.

7th. The conclusion that "motion" takes place in vegetables and cold-blooded animals, unattended by the development of heat, demonstrates that this peculiar motion does not agree with one of the laws of mobile bodies, and hence "motion" should not be the basis of a *physical* system of medicine.

8th. The conclusion that as "motion" is only the effect of a force, any system of medicine based on it precludes (for it is unnecessary) the consideration of the *cause* of the "motion;" and should we investigate the cause, we are without the limits of the basis; and as the tendency is to consider causes, it is unscientific to systemize on *effects*, in lieu of *causes*, as a basis in medicine.

9th. That the destructive changes observed in some diseases cannot be accounted for by "motion," for the *increased* circulatory speed, observed in some *active* maladies, is not *sufficiently* more rapid than the normal "motion" of a life-body, to account for the *rapid* waste. Thus, in acute dysentery, though there is rapid waste, "the pulse, in the majority of cases, is but little, and sometimes not at all, accelerated."*

10th. The conclusion that the effects of tonics take place without perceptible *increase* of normal organic "motion" (as iron upon the red globules), is opposed to this basis in medicine.

11th. The conclusion that emetics increase gastric "motion," but relax the cardiac sphincter, is opposed to this "motion"-basis, as such agents must act solely to increase or decrease "motion."

12th. The conclusion that the increased *motion* from stimuli, cathartics, diaphoretics, diuretics, and antispasmodics (as chloroform), fails to account for the *diminished motion* observed subsequent to their use; or the increased strength often resulting from sedatives, and always from natural sleep; and hence "motion" will not account for these phenomena, and should not constitute a medical basis.

13th. The conclusion that when astringents act mechanically, by coagulating fluids, the solid particles closing pores, the introduction of a "mechanical" quality is needed to render the action of such agents efficient, and shows that "motion" alone is insufficient as a basis in this relation.

14th. The conclusion that simple "motion" will not account for the fact that the effects of medicines *decrease* by use, when in physics the *same* force applied always produces an *equal* effect.

15th. That simple "motion" does not account for the *elective* affinity displayed by medicines on various organic bodies; nor for the effects of the same alkaloid, *e. g.*, of opium, when given to pigeons and man. Thus, narcotina, which has been taken by man, in doses of 120 grs., with impunity, when given to pigeons subcutaneously, in two grain doses, produces their death.†

It may also be stated here that therapeutic agents have, long since, been classified, on the basis of "motion," by the late lamented PROF. ROBLEY DUNGLISON.

These considerations seem to warrant the conclusion that a *scientific* system of medicine cannot be based on "motion."

Will a union of "form" and "motion," conjointly, enable us

* *Flint's Practice Medicine*, p. 351, third edition.

† See experiments of S. Weir Mitchell, M.D., *Hays' Journal*, January, 1870.

to found a *scientific* system of medicine? It is claimed these qualities will not enable us to do this, for the following reasons: The forces which produce organic "form" and organic "motion," are different in nature. The one is engaged in the particular *arranging*, the other in the *propulsion* of particles to be deposited. They are antipodal. The "form-force" develops a tendency to fixation, while "motion" presents a quality of destruction in the form. Suppose each of these "forces" could act in a right line — they would *always* continue *parallel*, unless *harmonized* by some intervening "force;" so, in an organic "form," these forces cannot *act as a unit* without the intervention of some *power*. Thus considered, "motion" and "form" cannot constitute the basis of a scientific system of medicine.

It seems obvious, too, that when either of two propositions are erroneous, the union of the two cannot establish truth; or "two wrongs will not make a right." Thus, if "form" and "motion," each viewed independently, does not develop respectively a scientific system of medicine, the union of these physical qualities will not accomplish this object, especially when it is shown that they cannot act as a unit without the operation of an intervening force. It would also seem well to remember here that, even did this "form" and "motion" system permit its author to call to his aid, in the erection of his doctrine, *all* the *physical* qualities of matter, it is necessary, to render the forces of matter harmonious in life, that there should be present a *positive independent force* to effect this unitarian result.

Three remarks of Dr. McElroy should be noticed:

1st. "Motion is the simple unit of force."

Motion being the result of force, and the latter being primary, it seems difficult to comprehend how "motion can be a unit of force;" *i. e.*, "a known determinate quantity, by the constant repetition of which any *other quantity* of the *same kind* is measured."* By this definition of *unus*, it can be understood that there may be a unit of "force," or of "motion," but it would seem impossible that one (force) can be a unit of the other, as they are *not* of the *same kind*.

2nd remark. "Our power over 'form' is *nil*, but our power over 'motion' is considerable."

* Olmstead's definition.

No one will deny the latter clause, but the former may be questioned. Organic forms being composed of particles, and constantly undergoing molecular changes, formative and destructive, it would be astonishing if such a form could not be influenced. Thus the general form of the body is influenced by external causes. A young and delicate boy enters the engineer corps, is exposed, has much exercise, eats and sleeps well therefrom, and grows rotund, large and solid, instead of the meagre boy. Again, certain Indian tribes flatten the heads of their children, that they remain so for life, and yet the brains of such distorted objects continue to perform their functions well. By the use of cod-liver oil we improve the *embonpoint* of our patients, and by the use of tonics we give power to digestion; and thence the nutritive system is improved, and the body enlarged. Starvation, too, diminishes the bulk of the body, and alcohol enlarges it. Further illustrations seem useless, but it seems proper to remember that what is here observed in *bulk*, takes place, in *minutiæ*, in organic bodies.

3rd remark. "Function depends on form." *Functio*, here the "office of any particular part of the animal body," is the result of "force," not of simple "figure." That the figure of a body may exercise some influence on neighboring parts (as pressure of one part against another, or by the interposition of organic substances) seems plain; but it is not supposed that such minor agencies give origin to the force which enables a part to perform a certain duty in animal life.

It would seem almost self-evident that the numerous organic structures, so similar in "form," yet performing such vastly different functions, would be an unanswerable argument against the doctrine that function depends on "form." That "form" may modify function, seems true; but it is claimed that "function" always depends on "force."

It remains to reply to the criticism of Dr. McElroy. In so doing, the peculiarities of life, as presented by myself, will be noticed as "Peculiarity," the criticism of the doctor as "Criticism," and the reply as "Rejoinder."

1st Peculiarity — "Activity, contra-distinguished from simple motion, and this presenting often, to our observation, as opposed to the operation of physical laws."

The above is an exact quotation from the original paper, and on which the following criticism was made. But, in the article as published, to express the idea more fully, the word "spontaneous" was prefixed to "activity."

"*Criticism:* Activity is a quality of motion; it is, in fact, motion — nothing more."

*Rejoinder:* "Activity" (spontaneous), from *ago*, "to drive gently or forcibly," implies the operation of some force which produces phenomena, and it embodies a quality of volition. It implies the presence of a power capable of setting bodies in motion, and bringing them to a state of rest. Motion is the simple propulsion of bodies, without power to change the direction of the propelled body. Motion is, therefore, passive. "Activity" (spontaneous) is volitional. Thus viewed, how can a volitional quality become a quality of simple motion, when the motion is the result of a volitional force, and only one of the phenomena of a life-body developing spontaneous "activity?" Or how can the greater be included in the lesser? It seems plain that motion may be a quality of activity, but not activity of motion, and that "activity" (spontaneous) is something more than mere motion.

2nd Peculiarity — "INDIVIDUALITY, which causes even like particles of matter to present different features."

"*Criticism:* The simplest element of individuality must be in form or forms."

*Rejoinder:* If admitted that "form" is the simplest element of individuality, such a definition would be imperfect, as it considers only the element "figure," neglecting even the force which gives origin to such organic peculiarities, and falling short of our mental conceptions of individuality, and dictating the conclusion that individuality is something more than "form."

3rd Peculiarity — "'REPRODUCTION,' or a perpetuation of INDIVIDUALITY."

"*Criticism:* "Reproduction, stated in its simplest terms, consists in form and motion."

*Rejoinder:* In the perpetuation of the individual, common and peculiar qualities of organic life are propagated. To claim, therefore, that these, in their "simplest terms," are "form" and "motion," ignores all the other qualities of life, which has already been shown in this article, is unscientific, and of no practical value, even were the statement admitted to be true.

4th Peculiarity — " IMMUTABILITY, or the maintenance of life, notwithstanding all the changes which take place in an organic body."

"*Criticism:* Maintenance of 'form,' with perhaps incessant, or perhaps frequent molecular changes." Also claims that organic bodies are not strictly immutable.

*Rejoinder:* It is claimed that beings can possess a *quality* of immutability, and not be strictly *immutable* — a quality possessed, in perfection, by the ALMIGHTY alone. Thus it was applied to organic bodies, to denote that while such structures were constantly undergoing molecular changes, the being still lived through this immensity of material change. It would also seem clear that in order that "form" may be maintained, with incessant molecular changes, something more than mere "figure" and "motion" is necessary that these qualities may be maintained.

5th Peculiarity — " SENSATION, or the appreciation of bodily states and wants."

"*Criticism:* That is, sight, hearing," etc. For the appreciation of these bodily states or wants, "a nerve mass or patch" is necessary, "and nerves and motion." "The simplest element of sensation is 'form' and 'motion.'"

*Rejoinder:* The statement that a "nerve patch" is necessary that sensation may take place, is well known. But this renders the subject no more clear, for it is still asked, what gives rise to the phenomena of sensation in the "nerve patch?" The "form" of the gray matter will not explain the mystery, for there are forms of gray matter, so far as figure is concerned, precisely like those in which this quality is developed; in which the phenomena of *motion* alone is observed, or motor and sensory centres. And, further, simple motion will not explain the peculiarities of sensation; and hence sensation cannot be accounted for on the basis of "form" and "motion."

6th Peculiarity—" The quality of PERCEPTION."

"*Criticism:* That is knowledge through the medium of bodily orgens, which must have 'form and motion' in their molecular structure, or perception can not take place."

*Rejoinder:* Even if admitted that *definite* "form" and motion is necessary, that perception may take place, it will be essential to prove how the simple "figure" of, or motion in, an organic body, can induce the diverse phenomena of Perception.

7th Peculiarity—" The quality of INSTINCT, and in man."

8th. The faculty of REASON.

9th. The faculty of CONSCIOUSNESS; and

10th. The WILL, and mental and moral phenomena in general.

"*Criticism:* These several qualities are each merged into 'form' and 'motion.' Instinct depends on 'form' and 'motion,' and intellect on added brain matter, having a definite and peculiar 'form,' and manifested by molecular motion. The other mental qualities here stated depend on added brain matter."

*Rejoinder:* The 7th, 8th, 9th and 10th peculiarities are here considered under one head, as they can all be met by one argument. Thus Dr. McElroy reduces them all to form and motion, and he associates them with "nerve patches."

Such peculiarities do not, of course, arise in bone or muscle. But because they are developed in nerve tissue, this fact does not explain the how these phenomena are produced; and unless this be solved (the latter), no new light is shed on what has already been learned by present systems; and hence the uselessness of adopting such a system as "form and motion." Again, this reduction ignores the qualities of matter in general, which is unscientific. Further, the assumption that reason, will, intellect, depend on brain matter, added to that amount of brain necessary to develop instinct, is refuted, from the simple fact that instinct relates to our bodily wants and protection, but the will, intellect, reason, it may be, to matters entirely foreign to the "physical." The one is occupied alone in protecting the individual, the other travels throughout the universe in its operations, and criticizes even the nature of this very "instinct." It is claimed that the simple multiplication of instinct can never produce, through "form" and "motion," the immensely varied phenomena, as to quality, observed in the will, reason and intellect.

While Doctor McElroy has shown much ingenuity in proposing and maintaining this "form and motion" system of medicine, and great boldness in the enunciation of his peculiar tenets, he has failed to convince the members of the "Muskingum County Medical Society" that his doctrines are founded in truth.

The great *desideratum* in the erection of a system of medicine based on physical laws, is *facts*, and these have not been, and must be furnished, ere our profession can be grounded upon physical data.

