# Addendum to Article of H. Culbertson, M. D.

**Published:** 1870-04

**Authors:** 


ARTICLE VI.—*Addendum to Article of H. Culbertson, M.D., Assistant Surgeon United States Army (Retired) in* THE CHICAGO MEDICAL JOURNAL *for March*, 1870.

IF "*form*" is understood to relate to "arrangement of particles," as well as to "figure," such a basis in medicine would not be a unit, as it would refer to "arrangement" and "figure." It would seem that the basis of any system of medicine should be a unit, and that there is no definition of "form" which can rightly and generally apply in the consideration of the magnitude, density, porosity, temperature, attraction, repulsion, elasticity, expansion, contraction, rest, statical and dynamical relations, weight, friction, hardness, resistance to fracture, endosmosis and exosmosis, capillarity, pneumatic relations, and to the origin and death of organic bodies. It would seem more definite and scientific to look at these several qualities directly, and not indirectly, through the medium of "form," which, thus employed, is at best but a mere classification, and serves to cover up the diverse relations and processes going on in an organic body.

The third division of my friend, of the principles operating in an organic body, is "form-force." This, it is claimed, is nothing but the old term "vitality" applied to "form," with the object of rendering the subject more clear, by or through "form." If the term "form-force" be applied to the whole animal body as a "form," then the parity in the expression "vitality" and "form-force," will be apparent, for each thus considered relates to the power which maintains the life-body entire. Thus viewed, "form-force" is "vitality;" but the "force" of this "form" is still a mystery, and the numerous phenomena which take place in such a "form" are no more comprehensible through the expression "form-force" than through the term "vitality."

ZANESVILLE, OHIO, *February* 15, 1870.

